# Absceso apical agudo y factores asociados en pacientes durante la pandemia de covid-19 en un centro de salud de acapulco, méxico

**DOI:** 10.21142/2523-2754-1202-2024-193

**Published:** 2024-06-27

**Authors:** Carlos Alberto Juárez-Medel, Eder Rodríguez-González, Ángel Neftalí Mendoza-Figueroa

**Affiliations:** 1 Departamento de Vinculación y Difusión en Estomatología de la Dirección General de Calidad y Educación en Salud, Subsecretaría de Integración y Desarrollo de la Secretaría de Salud Federal. Acapulco, Guerrero, México. carlos.juarez@salud.gob.mx Departamento de Vinculación y Difusión en Estomatología Dirección General de Calidad y Educación en Salud Subsecretaría de Integración y Desarrollo de la Secretaría de Salud Federal Acapulco, Guerrero México carlos.juarez@salud.gob.mx; 2 Departamento de Endodoncia de la Universidad Hipócrates. Acapulco, Guerrero, México. cd.eder.rodriguez@gmail.com Universidad Hipocrates Departamento de Endodoncia Universidad Hipócrates Acapulco, Guerrero Mexico cd.eder.rodriguez@gmail.com; 3 Centro de Endodoncia Especializada y Odontología Integral. Chilpancingo de los Bravos, Guerrero, México. nefrali88@gmail.com Centro de Endodoncia Especializada y Odontología Integral Chilpancingo de los Bravos, Guerrero México nefrali88@gmail.com

**Keywords:** endodoncia, expediente clínico electrónico, enfermedades periapicales, diente, México, endodontics, electronic clinical record, periapical diseases, tooth, Mexico

## Abstract

**Objetivo:**

Estimar la prevalencia de absceso apical agudo e identificar factores asociados en pacientes de un centro de salud de Acapulco, México.

**Material y métodos:**

Estudio epidemiológico que recopiló expedientes clínicos de pacientes durante octubre de 2021. Se recolectó información sociodemográfica, de vivienda, antecedentes personales patológicos, no patológicos y el registro del diagnóstico de la lesión periapical con base en sus características clínicas emitidas por el operador en turno. A través de un análisis multivariado, fue identificado un factor asociado al absceso apical agudo con la razón de momios y su intervalo de confianza del 95% como estimadores de la fuerza de asociación con el *software* estadístico CIETmap.

**Resultados:**

El 37% (32/87) de los pacientes fue diagnosticado con la infección. Respecto del sexo, las mujeres fueron las más afectadas con una proporción del 56% (18/32). La causa más común fue la caries con el 72% (23/32). El segundo premolar superior derecho fue el diente más afectado con el 25% (8/32). Se identificó un factor asociado, la edad de 36 a 62 años (RMa = 3,54; IC95%a = 1,27-16,62).

**Conclusión:**

La morbilidad reportada de la infección fue moderada y es una patología común en la consulta de urgencia estomatológica, por lo que es importante su manejo clínico por el profesional de área. Será importante implementar estrategias de educación para la salud bucal dirigidas a distintos grupos etarios con la finalidad de concientizar sobre el proceso cariogénico y sus consecuencias.

## Abstract (en)

**Objective:**

To estimate the prevalence of acute apical abscess and identify associated factors in patients of a health center from Acapulco, Mexico.

**Material and methods:**

Epidemiological study that collected clinical records of patients during the period of october 2021. Sociodemographic and housing information was collected, as well as personal pathological and non-pathological history and a record of the diagnosis of the periapical lesion based on its clinical characteristics issued by the operator on duty. Through multivariate analysis, a factor associated with acute apical abscess was identified with the odds ratio and its 95% confidence interval as estimators of the strength of association with CIETmap statistical software.

**Results:**

The 37% (32/87) of patients were diagnosed with the infection. In terms of gender, females were the most affected with a proportion of 56% (18/32). The most common cause was caries with 72% (23/32). The right upper second premolar was the most affected tooth with 25% (8/32). One associated factor was identified, age 36 to 62 years (ORa=3.54; CI95%a=1.27 -16.62).

**Conclusion:**

The morbidity rate of the infection was moderate, and it is a common pathology in stomatological emergency consultations, so it is important for the professional in charge of the area to manage it clinically. It will be important to implement oral health education strategies aimed at different age groups to raise awareness of the cariogenic process and its consequences.

## INTRODUCCIÓN

Las patologías pulpares consisten en la degeneración del tejido y se diagnostican por signos clínicos, evidencia radiográfica y sintomatología subjetiva del paciente, principalmente por dolor ^1^. La caries dental es la vía de entrada para los microorganismos y se correlaciona con la disminución de la fuerza de las paredes dentarias por afección de la dentina y con repercusión para el complejo pulpo-periapical ^2^. Una de estas patologías es el absceso apical agudo (AAA), que es una infección bacteriana en los tejidos de soporte periodontales. Se origina por vía pulpar y es causado por la progresividad de la caries al complejo pulpar y por periodontopatía, cuya etiología deriva de microorganismos aislados en bolsas periodontales. Evoluciona a una necrosis de inicio rápido, supuración, inflamación de tejidos asociados, y dolor espontáneo o a la percusión ^3^.

La distribución de las lesiones periapicales es variada en distintas poblaciones del mundo; por ejemplo, en Sudáfrica, la prevalencia de los abscesos dentales es del 15% en población de comunidades de ancestros étnicos con bajo nivel socioeconómico ^4^. En Sudamérica, se reporta una morbilidad del 44% al 55% en clínicas odontológicas de posgrado y universitarias de Venezuela y Colombia, en el orden dado ^(5, 6)^. Países del Caribe, como Cuba, reportan rangos de afección por AAA del 5% al 85% a nivel hospitalario ^7^^,^^8^. En México, algunos estudios en población de Yucatán mencionan registros del 2% al 30% ^9^^-^^11^.

Algunas investigaciones documentan condicionantes biológicas asociadas al AAA, donde las mujeres presentan mayor distribución de la infección ^5^^-^^7^, en comparación con los hombres ^8^^,^^9^. Respecto de factores modificables, como la edad, se reportan diferentes grupos etarios, de 35 a 59 ^5^^-^^9^ y de 60 a más años ^10^, por lo cual es evidente que la experiencia de caries antecede a la infección. Sobre los factores relacionados al autocuidado, se reporta a la dieta y la higiene oral deficiente ^7^^,^^8^. Asimismo, se describe la presencia de la infección en población portadora del virus de inmunodeficiencia humana y diabetes tipo 2 ^9^^,^^11^. Sobre los sectores de órganos más ocurrentes a la infección, se reportan los dientes anterosuperiores y los molares inferiores ^5^^,^^6^^,^^8^. 

La principal causa de aparición del AAA es el desarrollo progresivo de la caries dental no tratada ^5^^-^^9^. La manifestación de bacterias anaerobias en canales radiculares propicia el desarrollo infeccioso en el foramen apical ^12^. Si el sistema inmune no reacciona, la respuesta inflamatoria en los tejidos periapicales ocasiona necrosis pulpar ^13^. Se ha evidenciado presencia polimicrobiana en los abscesos, como *Fusobacterium nucleatum*, *Porphyromonas endodontalis*, *Parvimonas micra* y especies de *Streptococcus*. En órganos dentarios asintomáticos, hay predominio de *Porphyromonas endodontalis*, *Dialister invisus*, *Olsenella uli* y *Fusobacterium nucleatum*^14^. También se reportan especies de *Treponema socranskii*, *T. denticola*, *T. maltophilum* y *T. lecithinolyticum* en lesiones resistentes al tratamiento endodóntico de larga duración ^15^. Incluso, un estudio documentó la presencia de herpesvirus y papilomavirus en los abscesos apicales agudos con infección mayor a 5 mm del ápice radiográfico ^16^.

El AAA representa una urgencia estomatológica en los pacientes, por lo que debe tratarse de manera inmediata ^17^. Las complicaciones se presentan en los pacientes ante el avance de la infección a una etapa superior, como la celulitis odontógena ^18^^,^^19^. Las visitas periódicas al estomatólogo ayudan al diagnóstico precoz de infecciones odontogénicas ^7^. La fase I de esta patología constituye la más importante para diagnosticar y tratar, a fin de disminuir la gravedad de la infección, por lo que ofrece menos complicaciones del cuadro clínico ^17^.

En la región no existe evidencia sobre la morbilidad de esta patología. Además, durante el periodo de confinamiento derivado de la enfermedad por coronavirus de 2019 (COVID-19), se sugirió minimizar los procedimientos que generan gotas o aerosoles, y darle prioridad a aquellos que representan una urgencia, como el tratamiento del dolor severo por inflamación pulpar, la fractura de un diente vital y el trauma dental con avulsión/luxación ^20^^,^^21^. El objetivo de esta investigación fue estimar la prevalencia de absceso apical agudo e identificar factores asociados en pacientes de un centro de salud de Acapulco, México.

## MATERIAL Y MÉTODOS

Estudio epidemiológico de series de casos que recopiló información de expedientes clínicos de pacientes atendidos en un centro de salud del primer nivel de atención de Acapulco, México. Por conveniencia, fueron recolectados 95 expedientes clínicos de los primeros diez meses de consulta del año 2021. Los criterios de inclusión fueron expedientes clínicos de pacientes mayores de edad con los apartados debidamente llenados, con el diagnóstico presuntivo del operador en turno descrito y el seguimiento de monitoreo clínico. Para disminuir el sesgo de selección, fueron excluidos tres con diagnóstico de periodontitis, uno con absceso en tercer molar; y se eliminaron cuatro expedientes que no contaban con datos completos de los apartados de interés, por lo que la muestra final consideró 87 expedientes.

Del expediente clínico se tomaron datos sociodemográficos como sexo, edad, procedencia, estado civil, nivel de escolaridad, situación laboral, y los datos de la vivienda estimaron el nivel socioeconómico. Del apartado de antecedentes personales patológicos, se recolectó la presencia de enfermedades sistémicas. Del apartado de antecedentes personales no patológicos, se obtuvo información de la higiene oral (frecuencia de visitas al servicio dental, frecuencia de cepillado, uso de auxiliares en la higiene y conteo del biofilm). La variable dependiente fue la descripción del AAA anotada en el expediente clínico con base en el diagnóstico emitido por el operador de turno. El registro se tomó con una escala nominal, presencia y ausencia de la infección sobre el diente afectado con la nomenclatura de la Federación Dental Internacional ^22^.

Algunas variables politómicas, como el nivel socioeconómico, se estimaron de forma arbitraria con datos cercanos a su valoración, y se otorgaron puntajes a partir del material de las paredes de la vivienda, el número de cuartos para dormir y el tipo de piso ^23^. El índice de higiene oral fue obtenido con el registro de la nota médica de seguimiento, que tomó el conteo de biofilm con base en los parámetros de O’Leary. Para obtener el resultado, se usó un cociente cuyo numerador fue el total de caras pigmentadas, el denominador fue el total de caras presentes y el resultado se multiplicó por 100. Se dicotomizó en dos criterios nominales: biofilm oral ≤ 29% = higiene aceptable, y biofilm oral ≥ 30% = higiene deficiente ^24^.

Para evitar errores de digitación, se realizó una doble captación de datos y validación de estos con el *software* EpiData V3.1 ^25^. El análisis estadístico fue efectuado con el *software* CIETmap ^26^. Se llevó a cabo un análisis univariado para la obtención de frecuencias simples de las variables. Posteriormente, mediante tablas de contingencia, se efectuó un análisis bivariado mediante la razón de momios (RM) y su intervalo de confianza del 95% (IC95%) como medida de asociación entre potenciales factores con la variable dependiente a través del estadígrafo X^2^ de Pearson, con un límite de decisión ≤ 0,05. 

Finalmente, se elaboró un modelo de análisis multivariado explicativo de factores asociados al absceso apical agudo con el procedimiento Mantel-Haenszel ^27^. El modelo saturado inicial incluyó las variables significativas del análisis bivariado, ajustadas por aquellas consideradas por el criterio de plausibilidad biológica. Las variables fueron eliminándose una a una desde la asociación más débil, con método *backward*, hasta que quedaron aquellas con nivel de significancia ≤ 0,05 ^28^.

El protocolo de investigación fue aprobado por la Jefatura del Departamento de Estomatología de la Dirección General de Salud Municipal de Acapulco (México). El estudio no presentó ningún conflicto bioético, debido a que la información se obtuvo de registros secundarios y se consideró sin riesgo. Se resguardó la confidencialidad de los datos personales de identificación con base en lo establecido en la Norma Oficial Mexicana 004 de la Secretaría de Salud ^29^.

## RESULTADOS

El 54% (n = 47) de los pacientes fueron mujeres y el resto de hombres. El rango de edad osciló de 20 a 62 años, con una media de 44,2 ± 10,6. Sobre el lugar de procedencia, el 60% (n = 52) pertenecen a la zona rural y el resto, a la urbana. En la Tabla 1 se muestran en detalle las características sociodemográficas de la población.

**Tabla 1:** Información sociodemográfica en pacientes de un centro de salud de Acapulco, México

Características sociodemográficas	Categoría	n = 87	%
Sexo	Mujer	47	54%
	Hombre	40	46%
Lugar de procedencia	Rural	52	60%
	Urbana	35	40%
Estado civil	Soltero (a)	11	13%
	Casado (a)	45	52%
	Unión libre	10	11%
	Divorciado (a)	12	14%
	Viudo (a)	9	10%
Nivel de escolaridad	Sin estudios	10	12%
	Nivel básico	27	31%
	Nivel medio superior	30	34%
	Nivel superior	20	23%
Situación laboral	Desempleo	30	35%
	Trabajo informal	34	39%
	Trabajo formal	23	26%

Con base en las puntuaciones del hogar, el 60% (n = 52) fue de nivel económico bajo, el 37% (n = 32), del nivel medio, y una cifra ínfima, del nivel alto. Sobre las comorbilidades, el 60% (n = 52) las padece; la proporción de hipertensión arterial simultánea con diabetes se registró en el 58% (n = 30/52) y el resto fue diagnosticado con hipertensión (n = 22/52).

Del historial de visita odontológica, el 62% (n = 54) acudió a consulta dental hace dos o más años; el 21% (n = 18), hace un año; el 14% (n = 12), hace seis meses, y el resto nunca había acudido. La frecuencia de cepillado al día osciló de 1 a 6 veces, con una media de 3 ± 0,9. Con referencia al uso de auxiliares de higiene oral, el 51% (n = 44) usa al menos uno. Se registró el índice de biofilm de acuerdo con los parámetros de O’Leary, los cuales oscilaron entre el 16% y el 88%, con una media del 37% ± 14.

El 37% (n = 32) de los pacientes fue diagnosticado con AAA, en una proporción del 56% (n = 18/32) en mujeres y el 44% (n = 14/32) en hombres. La causa principal fue la existencia de caries, con el 72% (n = 23/32), seguida por los traumatismos. Respecto de los órganos dentales afectados, fueron los premolares los más recurrentes, con el 66% (n = 21/32), mientras que el 19% (n = 6/32) se presentó en los dientes anteriores y el 15% (n = 5/32) en los molares. El segundo premolar superior derecho fue el diente más relacionado con el AAA, con el 25% (n = 8/32) de los casos. En tanto a la automedicación, el 60% (n = 19/32) de los pacientes comentó haber usado medicamentos para las molestias. 

El análisis bivariado identificó dos factores asociados con la presencia de absceso apical agudo: la edad y el estado civil. La edad siguió una dirección en sentido de riesgo y el estado civil, en sentido de protección. La estimación no ajustada de asociación y el intervalo de confianza del 95% se muestran en la Tabla 2.

**Tabla 2:** Análisis bivariado de factores asociados al absceso apical agudo en pacientes de un centro de salud de Acapulco, México

Factor	Categoría	Absceso apical agudo		Sin absceso		RMna	IC95%	p
		n = 32	(%)	n = 55	(%)			
Sexo	Hombre ^(ref)^	14	16%	29	33%	0,71	0,29-1,68	0,420
	Mujer	18	21%	26	30%			
Edad	36-62 años ^(ref)^	30	34%	41	47%	5,12	1,21-21,71	0,030*
	20-35 años	2	3%	14	16%			
Lugar de procedencia	Rural ^(ref)^	21	24%	31	36%	1,48	0,60-3,66	0,390
	Urbana	11	13%	24	27%			
Estado civil	Sin pareja ^(ref)^	6	7%	26	30%	0,26	0,09-0,70	0,010*
	Con pareja	26	30%	29	33%			
Escolaridad	Sin estudios/nivel básico ^(ref)^	15	18%	22	25%	1,32	0,55-3,20	0,530
	Nivel medio superior/ superior	17	19%	33	38%			
Situación laboral	Desempleo/ trabajo informal ^(ref)^	24	27%	40	46%	1,12	0,41-3,06	0,810
	Trabajo formal	8	10%	15	17%			
Nivel socioeconómico	Bajo ^(ref)^	20	23%	32	37%	1,2	0,49-2,94	0,690
	Medio/alto	12	14%	23	26%			
Hipertensión	Sí ^(ref)^	22	25%	30	34%	1,83	0,73-4,59	0,190
	No	10	12%	25	29%			
Diabetes	Sí ^(ref)^	10	12%	20	23%	0,86	0,31-2,02	0,620
	No	22	25%	35	40%			
Visita odontológica	Nunca/2 o más años ^(ref)^	20	23%	37	42%	0,81	0,32-2,03	0,650
	Último año	12	14%	18	21%			
Frecuencia de cepillado	< 3 veces al día ^(ref)^	9	11%	15	17%	1,04	0,39-2,78	0,930
	≥ 3 veces al día	23	26%	40	46%			
Auxiliares de higiene oral	No Usa ^(ref)^	14	16%	29	33%	0,69	0,29-1,68	0,420
	Usa	18	21%	26	30%			
Biofilm	≥ 30% ^(ref)^	18	21%	32	37%	0,92	0,38-2,24	0,190
	≤ 29%	14	16%	23	26%			

RMna = Razón de momios no ajustada

IC 95% = Intervalo de confianza del 95%

p = Nivel de significancia de la X^2^ de Pearson

Ref = Categoría de referencia

Las dos variables que alcanzaron significancia estadística en el análisis bivariado fueron sometidas a un modelo saturado inicial multivariado, ajustados por las variables sexo y lugar de procedencia, por el criterio de plausibilidad. Solo la variable edad se mantuvo en el modelo final, por lo que demostró ser independiente en sentido de riesgo en los pacientes de 36 o más años. La fuerza de asociación ajustada con su intervalo de confianza del 95% se muestran en la Tabla 3. La prueba X^2^ de heterogeneidad fue mayor a 0,05 en la asociación del modelo final, por lo que se descarta un efecto distractor en el estrato. 

**Tabla 3:** Modelo final del análisis multivariado de factores asociados con el absceso apical agudo en pacientes de un centro de salud de Acapulco, México

Factor	RMa	IC95%a	X^2^ het	p
Edad: 36-62 años	3,54	1,27-16,62	0,122	0,880

RMa = Razón de momios ajustada.

IC95%a = Intervalo de confianza del 95% ajustado

X^2^ het = Chi cuadrada de heterogeneidad para identificar distractor de efecto

p = Nivel de significancia para la prueba de heterogeneidad

* El modelo saturado inicial incluyó la variable edad y estado civil; las cuales se ajustaron por sexo y lugar de procedencia

## DISCUSIÓN

Se encontró una prevalencia de AAA del 37%. La infección tuvo mayor distribución en las mujeres, con una razón 2:1 en comparación con los hombres. La edad resultó ser un factor de riesgo, pues los pacientes de 36 o más años tienen alrededor de tres veces más probabilidades de desarrollar la infección. 

Al ser un estudio de series de casos, los hallazgos solo son referentes de las personas que acuden al centro de salud, dado que no hay base poblacional, por lo que no es posible establecer diferenciaciones con la población de la región. El obtener datos de registros secundarios restringe la obtención de información de interés, lo que lleva a un sesgo de infrarregistro. Además, al ser reseñas del pasado, los datos recabados cambian después de la atención dental, por lo cual se evidencia el sesgo de previsibilidad retrospectiva. No obstante, el estudio identificó tendencias iniciales que sentarán las bases para seguir una línea de investigación de la temática en próximos estudios en la región.

Una desventaja de guiarse por datos ya asentados en los expedientes clínicos es que no se garantiza un diagnóstico fiable por parte de los operadores en turno del centro de salud. Esto debido a que, en los centros de salud del primer nivel de atención, la mayoría de los operadores tratantes no son endodoncistas, a lo cual, en ocasiones, se añade la falta de insumos que permitan emitir criterios clínicos más certeros. Lo que sí se garantiza es que los diagnósticos fueron establecidos con base en los códigos de la Clasificación Internacional de Enfermedades Aplicada a la Odontología y Estomatología, por lo que, normativamente, la codificación de la infección fue la apropiada en la descripción de los casos ^30^. En estudios que se realicen a futuro, será importante la obtención de datos mediante fuentes primarias, en donde los interexaminadores cuenten con el aval de endodoncistas, ya que son los más capacitados para emitir un diagnóstico fiable y diferencial de esta patología, y así minimizar el sesgo de observación mediante una estandarización del criterio diagnóstico.

La recopilación fue limitada, dado que se recolectaron expedientes clínicos durante un periodo de tiempo determinado, esto debido a que no fue posible obtener expedientes de años anteriores por cuestiones internas del centro de salud. El tamaño de muestra reducido se explica porque, durante la recolección de los expedientes, se atravesó un periodo donde la situación de la COVID-19 se mantuvo en semáforo rojo, por lo que la asistencia a consulta fue baja por las medidas del distanciamiento social y el miedo a infectarse. Cabe destacar que, entre los tratamientos catalogados como urgencia durante este periodo, fueron incluidos los de origen pulpar. 

La prevalencia de AAA que se halló fue moderada y estuvo dentro del rango reportado en investigaciones en población de Cuba (rango del 5% al 85%) ^7^^,^^8^. En comparación con estudios realizados en México, la morbilidad tuvo mayor distribución (rango del 2% al 30%) ^9^^-^^11^. Con respecto al diente afectado, el segundo premolar superior derecho fue el más ocurrente. Furzan ^5^ y Carmona-Lorduy *et al*. ^6^ mencionan que los dientes anteriores superiores son los más afectados en población venezolana y colombiana, respectivamente. Fernández-Collazo *et al*. ^8^ mencionan que son los molares, en la población atendida en servicios de urgencia de un policlínico de Cuba. No obstante, estos estudios son descriptivos, debido a que solo aportan la distribución del AAA y no documentan los factores asociados. 

Acerca del factor asociado de la edad, a mayor tiempo de exposición de los dientes a los ácidos producidos por las bacterias, mayor probabilidad de desarrollar caries. Datos similares en otras investigaciones describen esta variable como recurrente ^5^^-^^9^. Si bien algunos estudios sugieren la aparición de la infección en diferentes grupos de edad, suponemos que esto dependerá del autocuidado de la salud oral de cada persona, por lo que la patología puede desarrollarse en cualquier grupo etario ^5^^-^^10^. En los órganos dentarios cariados que no son tratados, la enfermedad progresa e invade el complejo pulpar, lo cual produce afecciones como el AAA; por ello, la edad predice la exposición progresiva del proceso carioso, lo cual antecede al efecto. 

Con relación a las condicionantes biológicas, como el sexo, el AAA tuvo mayor distribución en las mujeres, resultado similar al de investigaciones realizadas en clínicas de posgrado y universitarias de Venezuela y Colombia, respectivamente ^5^^,^^6^. Barberán-Díaz *et al*. ^7^ demostraron diferencias significativas en esta variable, y señalan una mayor predilección por este sexo en la población cubana. Por criterio de plausibilidad, ajustamos el modelo final multivariado con el sexo, sin encontrar diferencias significativas.

Un dato preocupante en el estudio fue que el 60% de los pacientes reportó un historial de automedicación por las molestias ocasionadas de la infección. Rôças ^14^ menciona que existe amplia variedad de microorganismos anaerobios en los abscesos. La automedicación es un grave problema que crea multirresistencia bacteriana, la cual es catastrófica y se estima que ocupará un lugar destacado en la agenda política mundial de atención de la salud ^31^^,^^32^. Por tanto, es importante que la farmacoterapia sea recetada por un profesional.

Recomendamos dos visitas periódicas al odontólogo cada seis meses, aunque no existan molestias, con el fin de una inspección rutinaria. Es necesario brindar información que impida que los pacientes recurran a la automedicación, puesto que eso repercutirá en su salud y economía a futuro. Los órganos dentarios que puedan conservarse deberían ser remitidos a terapia de conductos, y los de prognosis desfavorable, ser extraídos, con el fin de evitar complicaciones de la infección.

### Limitaciones

El obtener una muestra por conveniencia limita a que los resultados sean representativos de la población, por lo que no es posible extrapolarlos. Una característica del centro de salud es que se sitúa en la periferia de la ciudad, donde los usuarios, en su mayoría, son de zona rural, por ello no es posible comparar los resultados con la población que acude a los servicios públicos o privados de la zona urbana. Además, el centro de salud, al tratarse de un sitio de referencia, carece de base poblacional, por lo que los registros de la población incluida en el estudio no representan las características de la población de la región.

## CONCLUSIÓN

La prevalencia del AAA reportada en el estudio fue moderada y se identificó la edad como factor asociado al desarrollo de la infección. Este tipo de infección es común en la consulta de urgencia estomatológica, por lo que resulta importante su manejo clínico por parte del profesional de área. Una vez conocido el factor de riesgo, será necesario implementar estrategias de educación para la salud bucal dirigidas a distintos grupos etarios, con la finalidad de concientizar sobre el proceso cariogénico y sus consecuencias. 
